# Predicting antibody–antigen affinity with a dual-level representation model

**DOI:** 10.1093/bioinformatics/btag109

**Published:** 2026-03-10

**Authors:** Ziyang Wang, Yu Zhang, Youli Zhang, Jianwei Huang, Xiaoli Lu, Xiaoping Min, Shengxiang Ge, Jun Zhang, Ningshao Xia

**Affiliations:** Institute of Artificial Intelligence, Xiamen University, Xiamen, 361102, China; State Key Laboratory of Vaccines for Infectious Diseases, Xiang An Biomedicine Laboratory, School of Public Health; National Institute of Diagnostics and Vaccine Development in Infectious Diseases; National Innovation Platform for Industry-Education Integration in Vaccine Research; NMPA Key Laboratory for Research and Evaluation of Infectious Disease Diagnostic Technology, Xiamen University, Xiamen, 361102, China; Institute of Artificial Intelligence, Xiamen University, Xiamen, 361102, China; State Key Laboratory of Vaccines for Infectious Diseases, Xiang An Biomedicine Laboratory, School of Public Health; National Institute of Diagnostics and Vaccine Development in Infectious Diseases; National Innovation Platform for Industry-Education Integration in Vaccine Research; NMPA Key Laboratory for Research and Evaluation of Infectious Disease Diagnostic Technology, Xiamen University, Xiamen, 361102, China; State Key Laboratory of Vaccines for Infectious Diseases, Xiang An Biomedicine Laboratory, School of Public Health; National Institute of Diagnostics and Vaccine Development in Infectious Diseases; National Innovation Platform for Industry-Education Integration in Vaccine Research; NMPA Key Laboratory for Research and Evaluation of Infectious Disease Diagnostic Technology, Xiamen University, Xiamen, 361102, China; Institute of Artificial Intelligence, Xiamen University, Xiamen, 361102, China; State Key Laboratory of Vaccines for Infectious Diseases, Xiang An Biomedicine Laboratory, School of Public Health; National Institute of Diagnostics and Vaccine Development in Infectious Diseases; National Innovation Platform for Industry-Education Integration in Vaccine Research; NMPA Key Laboratory for Research and Evaluation of Infectious Disease Diagnostic Technology, Xiamen University, Xiamen, 361102, China; Information and Networking Center, Xiamen University, Xiamen, 361102, China; State Key Laboratory of Vaccines for Infectious Diseases, Xiang An Biomedicine Laboratory, School of Public Health; National Institute of Diagnostics and Vaccine Development in Infectious Diseases; National Innovation Platform for Industry-Education Integration in Vaccine Research; NMPA Key Laboratory for Research and Evaluation of Infectious Disease Diagnostic Technology, Xiamen University, Xiamen, 361102, China; School of Informatics, Xiamen University, Xiamen, 361102, China; State Key Laboratory of Vaccines for Infectious Diseases, Xiang An Biomedicine Laboratory, School of Public Health; National Institute of Diagnostics and Vaccine Development in Infectious Diseases; National Innovation Platform for Industry-Education Integration in Vaccine Research; NMPA Key Laboratory for Research and Evaluation of Infectious Disease Diagnostic Technology, Xiamen University, Xiamen, 361102, China; State Key Laboratory of Vaccines for Infectious Diseases, Xiang An Biomedicine Laboratory, School of Public Health; National Institute of Diagnostics and Vaccine Development in Infectious Diseases; National Innovation Platform for Industry-Education Integration in Vaccine Research; NMPA Key Laboratory for Research and Evaluation of Infectious Disease Diagnostic Technology, Xiamen University, Xiamen, 361102, China; State Key Laboratory of Vaccines for Infectious Diseases, Xiang An Biomedicine Laboratory, School of Public Health; National Institute of Diagnostics and Vaccine Development in Infectious Diseases; National Innovation Platform for Industry-Education Integration in Vaccine Research; NMPA Key Laboratory for Research and Evaluation of Infectious Disease Diagnostic Technology, Xiamen University, Xiamen, 361102, China

## Abstract

**Motivation:**

Protein language models are critical for modeling antibody–antigen interactions, yet sequence-based affinity prediction remains a key challenge, particularly when structural data are scarce. Existing methods often struggle to fully exploit sequence information, limiting their applicability across diverse antibody formats such as single-domain antibodies (sdAbs).

**Results:**

We propose dual-level protein representation for affinity prediction (DLP-Affinity), a dual-level deep learning framework for accurate sequence-based affinity prediction. It leverages two complementary modules: residue-to-residue to capture local interface contacts, and global stochastic projection embedding to represent global protein properties. Utilizing a fine-tuned protein language model, our approach achieves state-of-the-art performance on the general AB-Bind dataset (reducing mean absolute error by up to 20.9%) and delivers highly competitive results on the sdAb-DB dataset. This provides a robust tool for sequence-based antibody affinity prediction.

**Availability and implementation:**

The source code and datasets for DLP-Affinity are freely available at https://github.com/Zy-Wang-bit/DLP_Affinity and archived on Zenodo at https://doi.org/10.5281/zenodo.18437656

## 1. Introduction

Antibodies uniquely bind to antigens, neutralizing pathogens and toxins while marking them for immune response activation and complement system engagement. Beyond their natural roles, antibodies are pivotal in medicine and bioengineering as diagnostic markers (Zhou *et al.* 2024) and monoclonal drugs (Zinn *et al.* 2023) for targeted therapies. They also support vaccine development and diagnostic reagent preparation (Kevadiya *et al.* 2021). Current technologies for antibody preparation include polyclonal and monoclonal antibodies, along with surface display techniques that allow for large-scale antibody library construction. These methods rely heavily on empirical screening, whereas emerging computational techniques offer a more rational approach to design and selection.

In the field of protein engineering, computational methods have undergone a significant evolution, particularly with the advent of deep learning and large model techniques in recent years. Early computational methods based on statistical approaches (Smith and Waterman 1981) and machine learning (Wang *et al.* 2024b) were previously dominant, but performance and generalizability were limited by finite complexity. Deep learning has revolutionized protein sequence and structure modeling. It enables models to automatically extract complex features and to model protein–antigen interactions with high precision. A notable example is AlphaFold (Abramson *et al.* 2024), which leverages multiple sequence alignment, self-attention mechanisms, and graph neural networks to accurately predict protein 3D structures. Additionally, large-scale model architectures, such as evolutionary-scale modeling (ESM; Hayes *et al.* 2025), have gained traction in protein engineering, offering advanced antibody optimization techniques (Wang *et al.* 2024c) and enabling *de novo* design (Zheng *et al.* 2023). These innovations have significantly improved the precision and efficiency of antibody development, facilitating the creation of targeted therapies and vaccines.

Despite these advancements, evaluating generated antibodies remains challenging. Recent studies have employed approaches integrating sequence, structure, and surface information (Chen *et al.* 2023, Wu and Li 2023, Zhang *et al.* 2024), and atom-level representations (Jiale *et al.* 2024, Zheng *et al.* 2024) are pushing the field forward. However, sequence mining techniques remain underexplored, particularly where reliable structural data are unavailable.

Among various antibody formats, single-domain antibodies (sdAbs) present a compelling case for sequence-based modeling due to their small size, high stability, and unique structural features. The explosion of sdAb sequence data from high-throughput screening has created a scenario where sequence information vastly outpaces structural data, underscoring a critical need for computational methods that can effectively predict sdAb affinity from sequence alone.

In this paper, we propose dual-level protein representation for affinity prediction (DLP-Affinity), a novel framework that utilizes a protein language model (pLM) to organize amino acid sequence representations, and then generates feature representations at both the amino acid and protein levels. The pLM was fine-tuned on a sdAb sequence database to improve its ability to represent the features of sdAbs. Initially, the amino acid sequences of antigens and antibodies are input into the pLM to obtain initial feature vectors. For amino acid-level features, we employ an interaction representation module based on pLM features to generate new feature vectors. The global stochastic projection embedding (GSPE) module maps the pLM features into a fixed-dimensional global protein feature representation. The contributions of this work are as follows:

- We propose an effective dual-level protein feature representation mechanism, which improves performance on protein families with small datasets.
- Two feature representation modules, interaction representation module and GSPE, are introduced;
- We demonstrate the adaptability of our framework, showing that domain-specific fine-tuning of the pLM can significantly enhance performance on challenging scaffolds like sdAbs.
- Objective evaluations on datasets representing both general and sdAbs demonstrate the robustness and state-of-the-art performance of the proposed method.

## 2. Related work

### 2.1 Protein representation learning

Protein sequence representation learning has evolved from early statistical models to advanced embedding techniques. Previously, alignment-based methods (Ye *et al.* 2006) were used to extract informative features, but they often struggled to generalize across diverse protein families. Protein language models (pLMs, Brandes *et al.* 2022, Nijkamp *et al.* 2023, Hayes *et al.* 2025) have gained prominence by learning context-aware embeddings from large-scale sequence databases, which facilitate tasks ranging from structure prediction to *de novo* protein design (Zheng *et al.* 2023). Leveraging attention mechanisms, these models capture complex amino acid-level relationships.

Importantly, studies have demonstrated that pLM embeddings implicitly encode biophysically meaningful information beyond mere sequence patterns. Rives *et al.* (2021) showed that the ESM embedding space organizes amino acids by hydrophobicity, polarity, aromaticity, molecular weight, and charge. Subsequent work revealed that attention patterns in pLMs correlate with residue–residue contacts (Rao *et al.* 2021, Lin *et al.* 2023), and that pLMs can predict mutational effects on protein function in a zero-shot manner (Hayes *et al.* 2025). These findings suggest that statistical patterns learned from evolutionary data reflect underlying biophysical constraints, providing a theoretical foundation for sequence-only approaches to capture interaction energetics implicitly. These properties motivate our design, though we note that sequence-based representations inherently lack environmental context (see Conclusion and limitations).

While structure-based methods using graph neural networks (Jamasb *et al.* 2024) and atomic representations (Jiale *et al.* 2024, Zheng *et al.* 2024) have achieved remarkable progress, they require structural data that are often unavailable for novel antibody–antigen pairs. In contrast, sequence-based approaches remain essential for high-throughput screening scenarios where structural information is scarce or computationally expensive to obtain.

Recognizing the unique sequence and structural properties of antibodies, several specialized pLMs have been developed specifically for the antibody domain. Models such as AntiBERTy (Ruffolo *et al.* 2021), AbLang (Olsen *et al.* 2022), and IgLang (Leem *et al.* 2022) are trained exclusively on large corpora of antibody sequences. By learning the specific “grammar” of antibody variable regions, including the statistical constraints of complementarity determining region (CDR) loops and framework regions, these models have shown remarkable success in tasks like antibody numbering, developability prediction, and generation. While these specialized models are powerful, they are trained exclusively on antibody data. In contrast, our approach investigates the potential of adapting a large, general-purpose pLM (ESM2) to the antibody domain via fine-tuning. This strategy aims to leverage the broad biophysical knowledge learned from diverse proteins while specializing it for antibody–antigen recognition, a hypothesis strongly supported by our experimental results.

### 2.2 Antibody fitness evaluation

Antibody fitness prediction has evolved from statistical sequence motif analyses (Smith and Waterman 1981) to machine learning approaches (Yang *et al.* 2019, Dallago *et al.* 2022), which have provided more reliable fitness predictions (Rao *et al.* 2021, Hayes *et al.* 2025).

Molecular docking (Combs *et al.* 2013) and deep learning-enhanced docking (Zhang *et al.* 2023) also contribute to this field by considering the 3D structural information.

## 3. Materials and methods

### 3.1 Problem definition

We focus on predicting the antibody–antigen binding affinity (*K*_D_). Specifically, given the amino acid sequences of an antibody and an antigen as input, our goal is to develop a deep learning model that predicts the *K*_D_ as a continuous output. This is a supervised learning problem, which involves addressing two critical challenges:

- Challenge 1: Capturing global features. Standard sequence-based methods often process information locally, making it difficult to model long-range dependencies that define a protein’s overall fold and interaction surface.
- Challenge 2: Domain-specific adaptation. General-purpose pLMs, trained on millions of diverse protein sequences, may not capture the specific sequence patterns and structural constraints unique to antibody variable domains, potentially limiting their accuracy for affinity prediction.

Formally, the input space consists of antibody sequence $A=\{a_{1},a_{2},\ldots ,a_{m}\}$ and antigen sequence $G=\{g_{1},g_{2},\ldots ,g_{n}\}$, where $a_{i},g_{j}\in \{20\;\text{amino}\;\text{acids}\}$, with output space being the negative logarithm of the dissociation constant, $y=-{\;\text{log}\;}_{10}(K_{D})$ (commonly termed $pK_{D}$), where $K_{D}$ is in molar units (M). This logarithmic transformation is standard in the field for three reasons: (i) it linearizes the wide dynamic range of affinities (typically nanometer to micrometer); (ii) it enables direct comparison across datasets; and (iii) it relates to binding free energy via $\Delta G=2.303RT\cdot {\;\text{log}\;}_{10}(K_{D})$. For reference, a p*K*_D_ of 9 (*K*_D_=1 nM) corresponds to ΔG≈−12.3 kcal/mol, while a p*K*_D_ of 6 (*K*_D_=1 µM) corresponds to ΔG≈−8.2 kcal/mol at 298 K. Both benchmark datasets (sdAb-DB and AB-Bind) adopt this representation. The objective function minimizes the mean squared error (MSE) between predictions $\hat{y}=f_{\theta }(A,G)$ and ground truth:


(1) $$L=\frac{1}{N}\sum_{i=1}^{N}{\left({\hat{y}}_{i}-y_{i}\right)}^{2}.$$

### 3.2 Overview

**Figure 1:**
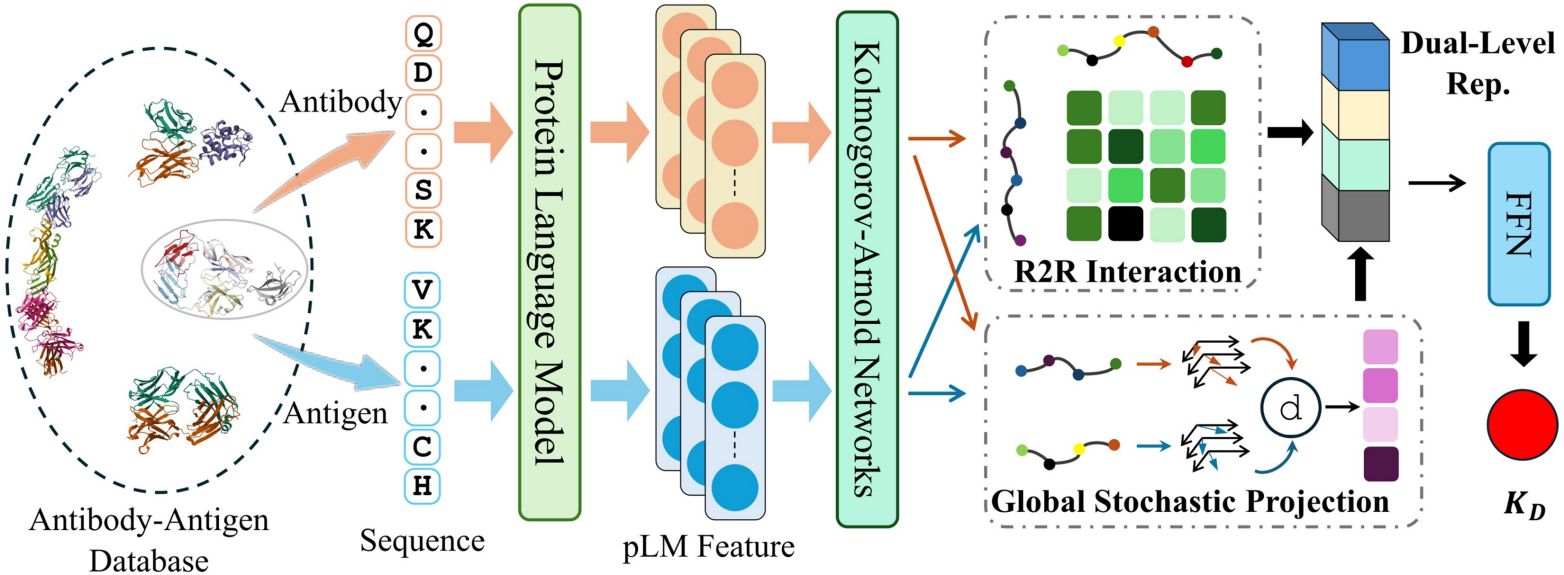
Framework of dual-level protein representation modeling. The R2R module captures local residue–residue contacts mediating direct binding interactions at the antibody–antigen interface, analogous to complementarity determining regions engaging epitopes. The GSPE module encodes global sequence properties reflecting overall protein fold and physicochemical characteristics, influencing binding specificity and stability beyond the immediate contact interface.

We start with an overview of our dual-level representation and prediction method (Fig. 1). The amino acid sequences of both the antibody and antigen are input into a protein language model, which generates context-aware residue-level features. These features are then reduced in dimensionality through Kolmogorov–Arnold networks (KANs), from an amino acid-level perspective, yielding initial feature representations for both the antibody and antigen. These initial embeddings are then processed by two parallel modules: (i) A residue-to-residue (R2R) interaction module computes a pairwise interaction matrix between antibody and antigen residues. (ii) A GSPE module converts each protein’s full sequence embedding into a single, fixed-dimensional vector. The resulting dual-level feature representations are then processed by a feed-forward network to produce the predicted outcomes.

The input features for both R2R and GSPE modules are residue-level embeddings extracted from our fine-tuned ESM2-3B model. For each amino acid position, ESM2 produces a 2560-dimensional context-dependent vector that encodes both the identity of the residue and its sequential context. Importantly, these are distributed representations learned from evolutionary patterns across millions of protein sequences, not hand-crafted features.

Differentiating from prior works, we propose hierarchical protein–amino acid fusion and antibody-specific fine-tuning strategies to enhance domain adaptation of pretrained models.

### 3.3 Fine-tuned pLM

**Figure 2:**
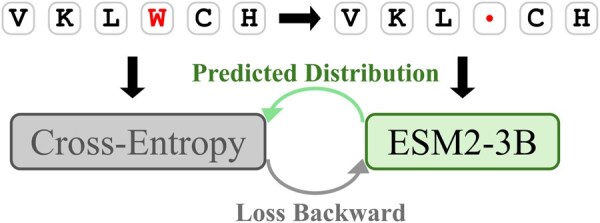
Fine-tuning of ESM2-3B.

Our framework leverages the pretrained ESM2-3B pLM (Lin *et al.* 2023) as the foundation for sequence representation learning. To enhance domain-specific understanding, we perform targeted fine-tuning using a curated database containing 11 228 599 antibody sequences (see Fig. 2).

During fine-tuning, we employ the masked language modeling objective, where 15% of amino acids in input sequences are randomly masked. The training objective minimizes the cross-entropy loss:


(2) $$L_{\mathrm{MLM}}=-\sum_{i\in M}\;\text{log}\;P(x_{i}|x_{\backslash M}),$$

where *M* denotes the set of masked positions and $x_{\backslash M}$ represents the observed context.

Our implementation introduces two key adaptations:

- CDR-prioritized masking: prioritizes masking in CDRs to focus on paratope-related patterns.
- Batch stratification: ensures balanced exposure to heavy and light chain variants through sequence-length-aware batching.

The model process sequences using learned embeddings of size 2560. Training utilized the AdamW optimizer with linear warmup over 40 steps (peak LR: 1e-5), achieving convergence in approximately 3 weeks on 16×A40 GPUs.

This fine-tuning phase allowed the base ESM2 model to specialize in antibody topology—particularly the conserved framework regions juxtaposed with hypervariable CDR loops—while maintaining its generalized understanding of protein biophysics from pretraining. We visualize the effect of fine-tuning on sequence representation using t-SNE analysis in Section 2.2, available as supplementary data at *Bioinformatics* online, demonstrating improved family-specific clustering (silhouette score: 0.44→0.68).

### 3.4 R2R interaction

This component establishes pairwise interaction patterns between antibody and antigen residues through a hierarchical feature transformation process, which consists of three sequential stages:

#### 3.4.1 Initial feature generation

We use the fine-tuned ESM2-3B model to extract initial residue-level embeddings for both antibody and antigen sequences. Let $X_{ab}\in R^{L_{ab}\times D}$ and $X_{ag}\in R^{L_{ag}\times D}$ represent the antibody and antigen feature matrices, respectively, where $L_{ab}$ and $L_{ag}$ are the sequence lengths, and D=2560 is the ESM2 embedding dimension. These high-dimensional features are then projected into compressed representations through stacked KANs (Liu *et al.* 2024), yielding reduced vectors $V_{ab}\in R^{L_{ab}\times 1}$ and $V_{ag}\in R^{L_{ag}\times 1}$. We hypothesize that the mapping from high-dimensional pLM embeddings to a residue’s interaction potential is highly nonlinear. KANs, based on the Kolmogorov–Arnold representation theorem, use learnable spline-based activation functions on edges rather than fixed activations on nodes. This architectural choice enables approximating a broader class of compositional functions with fewer parameters—advantageous when (i) the mapping has compositional structure and (ii) training data are limited, both conditions that apply to our affinity prediction task. We provide empirical validation comparing KAN with traditional MLPs in Table 5, available as supplementary data at *Bioinformatics* online, demonstrating consistent performance gains across both benchmark datasets.

#### 3.4.2 Interaction matrix construction

Pairwise residue interactions are computed via outer product of the compressed vectors:


(3) $$M_{\mathrm{inter}}=V_{ab}⊗V_{ag}^{⊤}\in R^{L_{ab}\times L_{ag}}.$$

Intuitively, this outer product operation can be understood as constructing a “contact map” between all antibody–antigen residue pairs. Each element $M_{\mathrm{inter}}[i,j]$ represents the interaction potential between the *i*th antibody residue and the *j*th antigen residue. By computing all pairwise combinations, we create a complete interaction space that subsequent layers can learn to distill—analogous to how molecular docking constructs an interaction grid.

We chose the outer product over attention mechanisms because: (i) as a parameter-free operation, it is robust against overfitting for limited datasets and (ii) it constructs a complete, unbiased interaction space encompassing all pairwise interactions, unlike attention which may overlook novel interaction modes. To preserve antibody-specific information, we concatenate *M*_inter_ with antibody features:


(4) $$M_{\mathrm{concat}}=\text{Concat}(M_{\mathrm{inter}},V_{ab})\in R^{L_{ab}\times (L_{ag}+1)}.$$

The concatenated matrix undergoes further refinement through additional KAN layers to distill interaction-aware residue representation $H_{AA}\in R^{L_{ab}\times 1}$. The residue representation captures positionwise interaction patterns while maintaining dimensional compatibility with downstream network components. Throughout this module, all KAN layers utilize sinusoidal activation functions with learnable coefficients, optimized through gradient-based adaptation during training.

### 3.5 Global stochastic projection embedding

Our GSPE module starts by transforming variable-length protein sequences into fixed-dimensional representations through stochastic geometric projections. Given an input residue embedding matrix $X\in R^{L\times d}$, for an amino acid sequence of length *L*, the embedding process consists of the following phases:

#### 3.5.1 Stochastic basis construction

Let *m* denote the number of projection vectors. We generate a random projection matrix:


(5) $$R={\left[r_{1}\|r_{2}\|\cdots \|r_{m}\right]}^{T}\in R^{m\times d},$$

where each row vector $r\in R^{d}$ is independently sampled from $N(0,I_{d})$. Columnwise $L_{2}$ normalization ensures unit hypersphere projection:


(6) $$r_{ij}\leftarrow \frac{r_{ij}}{\sqrt{\sum_{k=1}^{d}r_{ik}^{2}}}\;\forall j\in \{1,\ldots ,d\}.$$

#### 3.5.2 Projection and invariant encoding

For the *i*th projection vector, compute the sequence-level signature through:


(7) $$P_{i}=X\cdot r_{i}={\left[\langle x_{1},r_{i}\rangle ,\ldots ,\langle x_{L},r_{i}\rangle \right]}^{T}\in R^{L},$$

(8) $${\tilde{P}}_{i}=\text{sort}(P_{i})={\left[p_{\left(1\right)},p_{\left(2\right)},\ldots ,p_{\left(L\right)}\right]}^{T}.$$

This sorting operation ensures permutation invariance while maintaining relative magnitude relationships. While this discards local sequence order, the pLM embeddings already capture sequential context, and GSPE’s role is to derive a global, order-invariant signature. The final embedding is formed by concatenating all *m* projections:


(9) $$h=\left[{\hat{p}}_{1}\;⊕\;{\hat{p}}_{2}\;⊕\cdots ⊕\;{\hat{p}}_{m}\right]\in R^{m}.$$

#### 3.5.3 Multiprojection aggregation

For antibody–antigen pair (A,B), we generate independent projection sets $P_{A_{i=1}^{n}}^{i}$ and $P_{B_{i=1}^{n}}^{i}$. The interaction feature derives from:


(10) $$d^{\left(i\right)}=\sqrt{\sum_{j=1}^{m}{\left(\frac{{\hat{p}}_{A,j}^{\left(i\right)}-{\hat{p}}_{B,j}^{\left(i\right)}}{\sigma_{j}}\right)}^{2}},$$

where $\sigma_{j}$ represents trainable scaling parameters. The final protein-level embedding:


(11) $$h_{\text{pair}}=\left[\Phi (d^{\left(1\right)})\;⊕\cdots ⊕\;\Phi (d^{\left(n\right)})\right]\in R^{n},$$

with $\Phi (x)=\text{log}(1+e^{x})$ ensuring positive-definite outputs.

The sorted projections ensure permutation invariance: ${\hat{p}}_{i}(\Pi X)={\hat{p}}_{i}(X)$ for any permutation Π. We provide qualitative analyses validating GSPE’s permutation invariance and the effect of projection count *m* on embedding stability in Section 2.1, available as supplementary data at *Bioinformatics* online.

## 4. Results

### 4.1 Ablation study

We systematically evaluate the contributions of the R2R (R2R interaction) and GSPE modules through ablation experiments (Table 1). The ablation reveals complementary module roles: on sdAb-DB, R2R contributes more significantly (MAE: 0.174→0.267 without R2R versus 0.174→0.216 without GSPE), reflecting local pairwise contacts’ importance for compact sdAb binding interfaces. Conversely, on AB-Bind, GSPE shows comparable importance (MAE: 0.136→0.186 without R2R versus 0.136→0.162 without GSPE), indicating global properties become critical for diverse antibody formats. The full model achieves optimal performance by integrating R2R’s fine-grained interaction modeling with GSPE’s global sequence abstraction, jointly optimizing local and global protein representations.

**Table 1:** Ablation experiments.

Dataset	Method	MAE	RMSE	$R^{2}$	PCC
sdAb-DB	w/o R2R	0.267	0.347	0.412	0.645
	w/o GSPE	0.216	0.293	0.495	0.710
	DLP-Affinity	0.174	0.253	0.586	0.760
AB-Bind	w/o R2R	0.186	0.267	0.592	0.544
	w/o GSPE	0.162	0.246	0.616	0.580
	DLP-Affinity	0.136	0.233	0.641	0.649

### 4.2 Benchmark results

Our proposed model, particularly the variant utilizing the fine-tuned ESM2 model (ESM-FT), establishes a new state-of-the-art performance across the two benchmark datasets when considering a holistic view of the evaluation metrics (Table 2). Notably, our larger gains on AB-Bind (20.9% MAE reduction versus DeepNano-seq) compared to sdAb-DB (3.3%) likely reflect the relative maturity of methods on each benchmark: on sdAb-DB, multiple approaches already achieve MAE near 0.17–0.18, leaving limited room for improvement, whereas AB-Bind presents greater headroom for gains. Additionally, AB-Bind’s structural diversity may favor our dual-level representation.

**Table 2:** Performance comparison of different models.

	sdAb-DB				AB-Bind			
Model	MAE	RMSE	$R^{2}$	PCC	MAE	RMSE	$R^{2}$	PCC
ab-predictor (Kurumida et al. 2020)	0.253	0.310	0.524	0.570	0.263	0.370	0.324	0.413
AttABseq (Jin et al. 2024)	0.258	0.370	0.362	0.440	0.370	0.294	0.348	0.360
Antiformer (Wang et al. 2024a)	0.208	0.280	0.535	0.620	0.220	0.259	0.427	0.483
MVSF-AB (Li et al. 2025)	0.175	0.260	0.540	0.738	0.180	**0.220**	0.450	0.520
DeepNano-seq (Deng et al. 2024)	0.180	**0.251**	**0.591**	**0.765**	0.172	0.241	0.556	0.633
DLP-Affinity (original ESM)	0.195	0.281	0.537	0.687	0.233	0.570	0.414	0.495
DLP-Affinity (ESM-FT)	**0.174**	0.253	0.586	0.760	**0.136**	0.233	**0.641**	**0.649**

Bold values indicate the best performance for each metric.

On the sdAb-DB dataset, our model demonstrates performance that is highly comparable to the strongest baseline, DeepNano-seq. While DeepNano-seq shows a marginal advantage in RMSE (0.251 versus 0.253), $R^{2}$ (0.591 versus 0.586), and PCC (0.765 versus 0.760), our model achieves a lower (better) MAE of 0.174 compared to DeepNano-seq’s 0.180. It is crucial to contextualize this result: DeepNano-seq is an ensemble framework that integrates multiple feature types (including PSSM) and model architectures (CNN, Bi-LSTM) to make its final prediction. In contrast, our approach relies on a single-model architecture operating purely on sequence information derived from a pLM. Achieving such competitive performance without the added complexity of model ensembling or engineered features highlights the efficacy and elegance of our dual-level representation scheme.

On the AB-Bind dataset, our model demonstrates superior generalization and robustness, outperforming all baseline methods on the majority of key metrics. It achieves a substantial 20.9% reduction in MAE compared to the best baseline (0.136 versus DeepNano-seq’s 0.172) and a significant 15.3% increase in the coefficient of determination ($R^{2}$) (0.641 versus DeepNano-seq’s 0.556). This indicates our model explains a much larger portion of the variance in the data. While the MVSF-AB model maintains a slightly lower RMSE (0.220 versus our 0.233), our model’s commanding performance across MAE, $R^{2}$, and PCC underscores its strong predictive power on this more diverse dataset.

We also performed preliminary validation using deep mutational scanning data for the LY-CoV555 antibody (PDB: 7KMG), an independent test case not present in training data. The model showed moderate ability to distinguish binding-disrupting mutations from neutral ones with an area under the receiver operating characteristic curve (AUC) of 0.85, though quantitative prediction accuracy was limited by data imbalance (see Section 2.3, available as supplementary data at *Bioinformatics* online, for details and caveats).

## 5. Conclusion

This work introduces DLP-Affinity, a dual-level protein representation framework for antibody affinity prediction that integrates residue-level interaction modeling (R2R) with global sequence semantics (GSPE) upon fine-tuned pLM embeddings. Importantly, our framework achieves state-of-the-art performance using a single, unified model operating purely on sequence information. Unlike ensemble approaches that require combining multiple feature types (PSSM, structure predictions) and model architectures, DLP-Affinity offers a simpler and more deployable solution for high-throughput antibody screening pipelines. On two benchmark datasets, we demonstrate up to a 20.9% reduction in MAE and a 15.3% $R^{2}$ improvement over strong baseline methods, with systematic ablation studies confirming the complementary contributions of both modules.

Despite these advancements, four limitations warrant further investigation. First, generalizability to multichain antibodies (e.g. IgG) remains unvalidated. The R2R module linearizes sequences and may not capture complex spatial interactions between noncontiguous heavy and light chains. Two extensions are feasible: (i) concatenating the heavy and light chain variable domains (VH-VL) with a separator token or (ii) graph-based modeling with interchain edges, which may better preserve the paratope’s spatial organization. Second, our approach relies solely on sequence-derived features. Incorporating structural information—for instance, replacing the R2R’s outer product with a graph neural network operating on AlphaFold3-predicted contact maps (edges at <8 Å)—could enhance physical interpretability by leveraging structural priors. Third, sequence-based models inherently lack environmental context. Affinity is modulated by pH, ionic strength, and posttranslational modifications (e.g. glycosylation), which are absent from standard sequence inputs. Finally, although our predictions are statistically significant, experimental validation via surface plasmon resonance or bio-layer interferometry is necessary to bridge the gap between computational predictions and measured *K*_D_ values.

Notwithstanding these limitations, our work establishes a principled framework for protein representation learning. This methodology provides a robust foundation for advancing computational antibody engineering and integrating affinity prediction into generative design pipelines.

## Supplementary Material

btag109_Supplementary_Data

## Data Availability

The datasets analyzed during the current study are all publicly available. The sdAb-DB is available as described in Wilton *et al.* (2018). The AB-Bind dataset can be accessed as detailed in Sirin *et al.* (2016). The large-scale nanobody sequence dataset (approximately 11M sequences) from the INDI project (NaturalAntibody), used for fine-tuning the ESM model, is publicly available for academic research purposes on https://research.naturalantibody.com. Users should refer to the original data provider’s terms of use for commercial applications. The source code for our model is available on GitHub (https://github.com/Zy-Wang-bit/DLP_Affinity) with an archival version deposited on Zenodo (DOI: https://doi.org/10.5281/zenodo.18437656) under the MIT license for unrestricted reuse.
