# Supplementary material for: Predicting antibody–antigen affinity with a dual-level representation model
Source: Bioinformatics. 2026 Mar 10;42(4):btag109. doi: 10.1093/bioinformatics/btag109 (PMC13070686; doi:10.1093/bioinformatics/btag109)
Supplement: btag109_Supplementary_Data [file btag109_supplementary_data.pdf]

Supplementary Material for:

Predicting Antibody-Antigen Affinity with a Dual-Level Representation Model

Ziyang Wang, Yu Zhang, Youli Zhang, Jianwei Huang, Xiaoli Lu,
Xiaoping Min, Shengxiang Ge, Jun Zhang, and Ningshao Xia

January 31, 2026

Contents

S1 Experimental Configuration **2**
S1.1 Datasets 2
S1.2 Baseline 2
S1.3 Evaluation Metrics 3

S2 Supplementary Results **3**
S2.1 Qualitative Analysis of the GSPE Module 3
S2.1.1 Permutation Invariance Validation 3
S2.1.2 Impact of Multiple Projection Count 5
S2.2 Effect of Fine-tuning ESM2 5
S2.3 Independent Validation on LY-CoV555 DMS Data 6
S2.3.1 Data Preprocessing 7
S2.3.2 Results 7
S2.4 KAN vs MLP Ablation Study 8

S1 Experimental Configuration

S1.1 Datasets

The datasets utilized in this study include the Single Domain Antibody Database (sdAb-DB) [1] and AbBind [2]. The sdAb-DB, which is derived from camelid antibodies, comprises 1,446 sequences gathered from literature sources, the Protein Data Bank (PDB), and the National Center for Biotechnology Information (NCBI). Additionally, 290 antibody-antigen pairs were selected for validation experiments. AbBind, which primarily focuses on antibody binding data, contains over 1,100 interactions between antibodies and antigens. This dataset provides experimentally validated information regarding binding specificity, affinity, and functional characteristics. Together, these datasets serve as valuable resources for the investigation of antibody-antigen interactions.

ESM Fine-tuning Dataset: To fine-tune the ESM models for improved representation of single-domain antibodies, we employed the NGS nanobody sequences dataset [3] from INDI. This large-scale dataset contains approximately 11,228,600 unique single-domain antibody sequences. For the fine-tuning process, this dataset was partitioned into training, validation, and test sets using a 90:5:5 ratio.

Baseline Datasets Splitting: To ensure a rigorous evaluation of model generalization and prevent data leakage from highly similar sequences, the partitioning of the sdAb-DB and AbBind datasets was not performed randomly on individual protein pairs. Instead, we adopted a family-aware splitting strategy. First, sequences were grouped into families, where each family consists of a set of mutants derived from a single wild-type protein. The split into training, validation, and test sets was then performed at the level of these families, using an 8:1:1 ratio. This ensures that all mutants originating from the same wild-type protein are contained within the same data split (e.g., training), preventing the model from being tested on sequences that are trivial variations of what it has seen during training. For smaller protein families with fewer than 10 members, we implemented a strategy to guarantee that at least one member was allocated to the validation set and at least one to the test set, ensuring that all families are represented across the evaluation pipeline.

S1.2 Baseline

For baseline comparison, we utilized models designed for predicting antibody-antigen binding, encompassing both qualitative interaction (presence/absence) and, where applicable, quantitative affinity. The first baseline model is ab-predictor [4], an ensemble model that integrates predictions from software tools such as Rosetta and FoldX, both well-established docking tools for simulating molecular interactions and predicting affinity. Additionally, we incorporated four state-of-the-art sequence-based models: AttABseq [5], which employs attention mechanisms; AntiFormer [6], based on transformer architecture; MVSF-AB [7], which utilizes multi-view sequence features; and DeepNano-seq [8], an ensemble deep learning framework capable of predicting general protein-protein interactions (PPI) and nanobody-antigen interactions (NAI) directly from sequence. While DeepNano-seq provides an interaction score indicating the likelihood of binding, it is primarily optimized for interaction prediction rather than quantitative affinity estimation. Together, these models offer a comprehensive comparison, merging traditional docking methods with cutting-edge deep learning advancements in sequence-based prediction.

S1.3 Evaluation Metrics

To comprehensively evaluate the performance of our antibody-antigen binding affinity prediction model, we employ four widely-adopted regression metrics:

$$\mathrm{MAE} = \frac{1}{n}\sum_{i=1}^{n}|y_i - \hat{y}_i|, \tag{S1}$$

$$\mathrm{RMSE} = \sqrt{\frac{1}{n}\sum_{i=1}^{n}(y_i - \hat{y}_i)^2}, \tag{S2}$$

$$R^2 = 1 - \frac{\sum_{i=1}^{n}(y_i - \hat{y}_i)^2}{\sum_{i=1}^{n}(y_i - \bar{y})^2}, \tag{S3}$$

$$\mathrm{PCC} = \frac{\sum_{i=1}^{n}(y_i - \bar{y})(\hat{y}_i - \bar{\hat{y}})}{\sqrt{\sum_{i=1}^{n}(y_i - \bar{y})^2}\sqrt{\sum_{i=1}^{n}(\hat{y}_i - \bar{\hat{y}})^2}}, \tag{S4}$$

where y, $\hat{y}$, and $\bar{y}$ denote the experimental value, predicted value, and mean of experimental K_D measurements, respectively.

MAE and RMSE quantify prediction errors at distinct scales: while MAE reflects absolute deviations, RMSE exhibits higher sensitivity to larger errors through quadratic penalization – particularly relevant for biological data where extreme deviations may indicate biologically significant discrepancies. R^2 measures the proportion of variance explained by the model, with 1 indicating perfect variance capture. PCC evaluates the linear correlation between predictions and experimental values, a critical criterion for validating regression consistency in affinity prediction tasks.

S2 Supplementary Results

S2.1 Qualitative Analysis of the GSPE Module

To gain a deeper understanding of the GSPE module's characteristics, we conducted a series of qualitative analyses. These experiments investigate its robustness to sequence permutations, its ability to capture structural information of protein families, and the impact of the key hyperparameter, the number of projections m.

S2.1.1 Permutation Invariance Validation

One of the core design principles of the GSPE module is its invariance to local permutations in the input sequence. To validate this property, we selected a protein sequence and randomly shuffled the order of amino acids within a small, non-critical region. Subsequently, we compared the cosine similarity between the embedding vector h generated by the GSPE module for the original sequence and the perturbed sequence. As illustrated in Figure S1, the GSPE module maintained an extremely high cosine similarity (approaching 1.000) between the original and perturbed embeddings, indicating its ability to effectively disregard such local permutation changes and focus on broader sequence composition. In contrast, under the same level of perturbation, traditional sequence models (e.g., direct embeddings from LSTM or Transformer) exhibited significantly larger differences in their embeddings (e.g., a cosine similarity around 0.690), highlighting their sensitivity to sequence order. This result strongly demonstrates the robustness of the GSPE module when handling local sequence rearrangements.

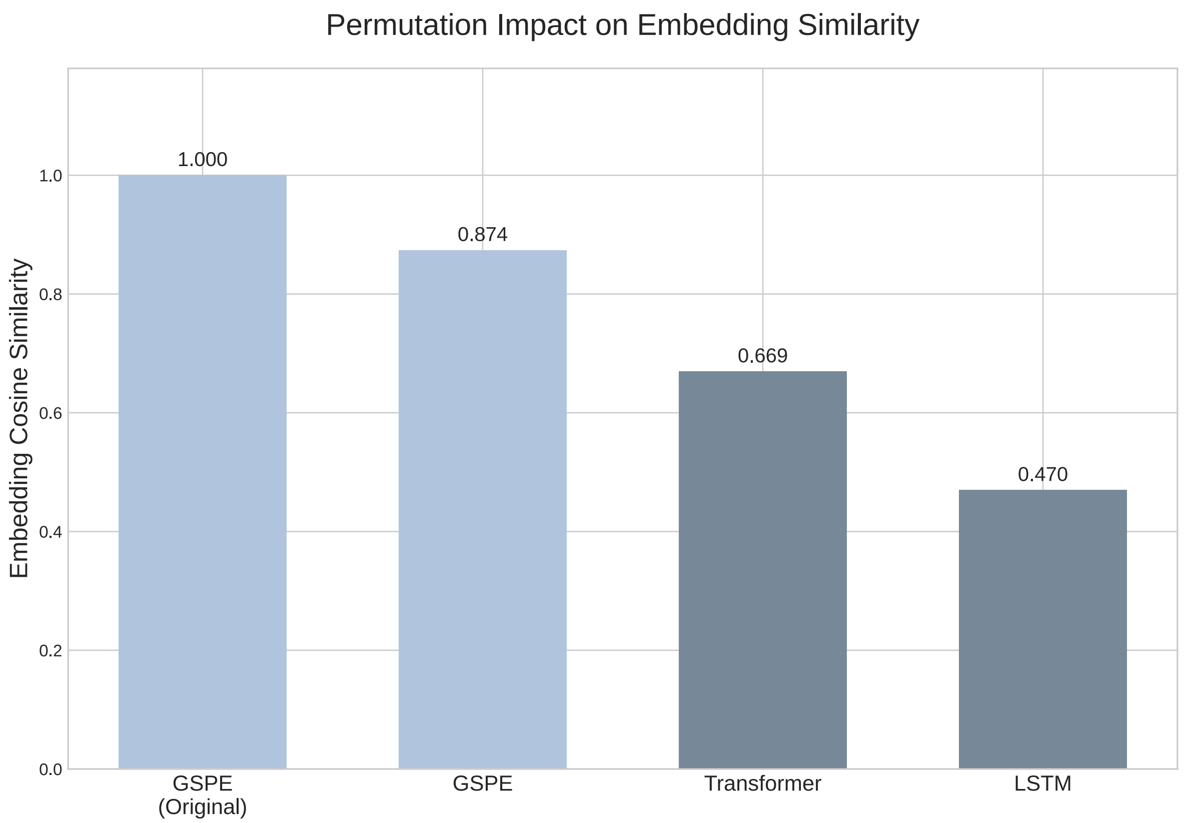

Figure S1: Permutation invariance validation of the GSPE module. The left bar shows that GSPE maintains a cosine similarity close to 1.000 for embeddings after local sequence perturbation. The right bar represents a baseline method, shows a significantly reduced similarity (e.g., 0.690) under the same perturbation.

S2.1.2 Impact of Multiple Projection Count

The GSPE module incorporates a multiple projection mechanism, controlled by the parameter m (number of projections). The choice of m is crucial for the quality and stability of the resulting embeddings. An overly small m might lead to insufficiently specific feature representations, while an excessively large m could incur unnecessary computational overhead. To investigate the impact of m, we compared the distribution of cosine similarities between multiple independent embeddings of the same protein, generated using different values of m (e.g., m=10 for fewer projections and m=128 for more projections). As depicted in Figure S2, when m=10, the cosine similarity distribution of multiple embeddings was more dispersed with a lower median, suggesting less stable and consistent results. In contrast, increasing m to 128 resulted in a highly concentrated distribution with a median close to 1.0, indicating that a sufficient number of projections enables GSPE to produce highly stable and consistent protein embeddings. This analysis aids in selecting a reasonable m value that balances embedding quality with computational efficiency for a given task.

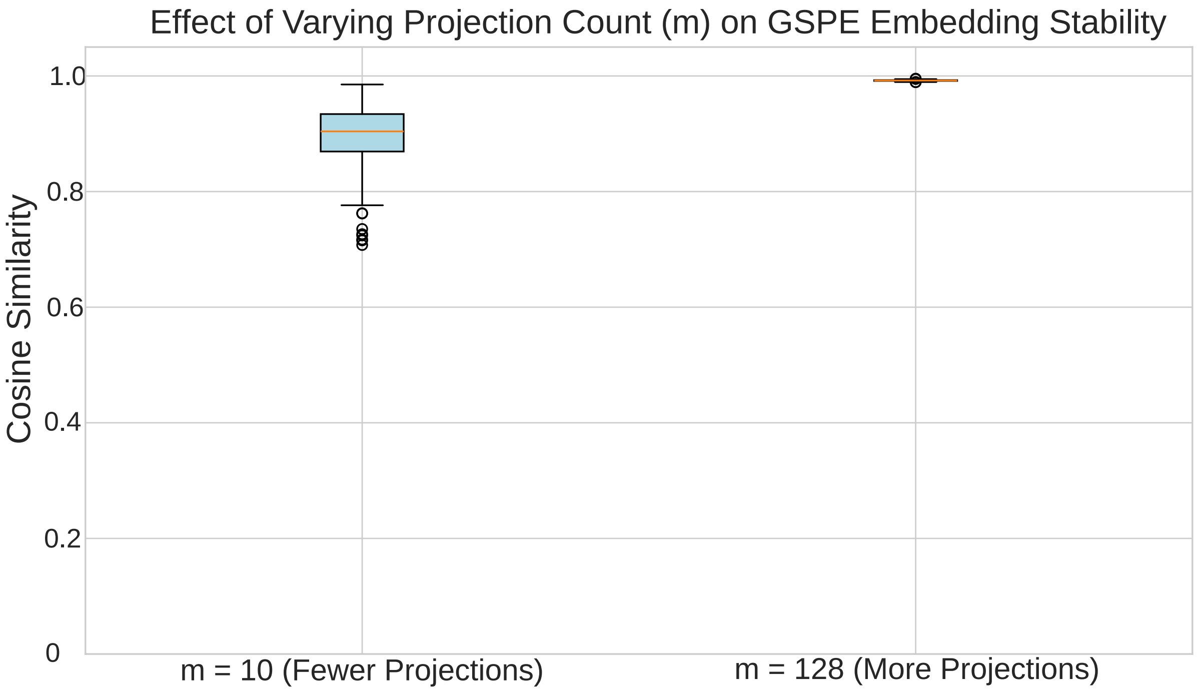

Figure S2: Effect of varying projection count (m) on GSPE embedding stability. The box plots show the cosine similarity distribution of multiple embeddings for the same protein using m=10 (fewer projections) and m=128 (more projections). Higher and more stable similarities are observed for m=128.

S2.2 Effect of Fine-tuning ESM2

To validate the effectiveness of fine-tuning, we systematically compared the original ESM2 with our fine-tuned model (ESM2-FT) on affinity prediction tasks. As shown in Table S1, ESM2-FT achieves 10.6% and 30.7% higher Pearson correlation in two benchmark datasets compared to the original model, demonstrating significant improvements in capturing antibody-antigen interaction patterns.

We further analyzed the pLM representations using t-SNE visualization to illustrate how fine-tuning enhances the model's ability to discriminate protein functional families (Fig. S3). The

Table S1: Performance comparision of different models.

	Sdab-db				Abbind			
DLP-Affinity (Original ESM)	0.195	0.281	0.537	0.687	0.233	0.570	0.414	0.495
DLP-Affinity (ESM-FT)	**0.174**	0.253	0.586	0.760	**0.136**	0.233	**0.641**	**0.649**

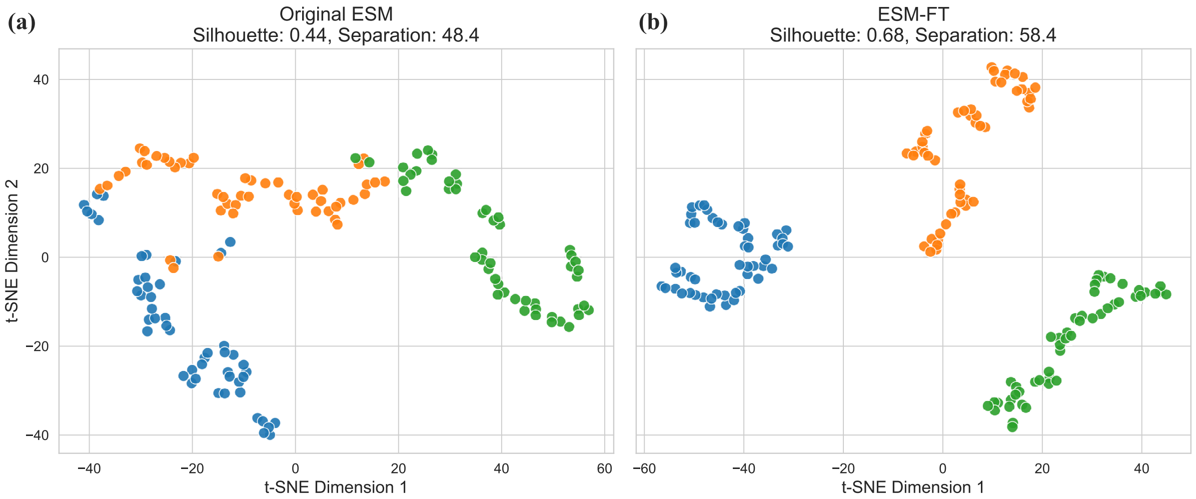

Figure S3: Antibody sequence homology visualization by t-SNE.

improved silhouette score (0.44 → 0.68) and inter-cluster separation (48.4 → 58.4) quantitatively confirm that ESM2-FT learns more family-specific representations. After fine-tuning, the t-SNE plot (Fig. S3b) shows the emergence of distinct subclusters within each protein family. This observation suggests that the fine-tuned model is learning to distinguish between closely related sequences. We hypothesize that these learned features correspond to subtle variations, such as those in the paratope, that are critical for determining binding affinity.

These subclusters likely correspond to evolutionary trajectories and structural micro-variations that influence affinity. The hierarchical feature separation aligns with biophysical principles—gross family differences govern baseline binding potential, while subcluster-level features modulate affinity magnitudes. This provides an explanation for ESM2-FT's superior performance in the affinity prediction task (Table S1).

S2.3 Independent Validation on LY-CoV555 DMS Data

To validate the model's capability in predicting mutational effects on antibody-antigen binding, we utilized independent deep mutational scanning (DMS) data for the LY-CoV555 (bamlanivimab) antibody [9]. LY-CoV555 is a therapeutic neutralizing antibody targeting the SARS-CoV-2 spike protein receptor-binding domain (RBD); its crystal structure is available in the PDB (accession 7KMG).

The DMS dataset quantifies escape fractions for single-amino-acid mutations across the RBD (residues 331–531), where higher fractions indicate reduced binding. This dataset was selected for two primary reasons:

1. **Temporal Independence:** PDB 7KMG was deposited in January 2021, postdating the publication of our training benchmarks (sdAb-DB, 2018; AbBind, 2016), ensuring no data leakage.

2. **Clinical Relevance:** Validation on a therapeutic antibody like LY-CoV555 enhances the practical significance of the model's performance.

S2.3.1 Data Preprocessing

Sequence Preparation: The antibody sequence was extracted from PDB 7KMG, consisting of the variable heavy (VH, 119 residues) and variable light (VL, 107 residues) chains. These were concatenated into a single 226-residue input string. The wild-type RBD sequence (211 residues, Wuhan-Hu-1 reference) served as the antigen template.

Stratified Down-sampling: The raw DMS dataset exhibited severe class imbalance, with specific escape scores (particularly low values) dominating the sample distribution. To mitigate the bias introduced by this skewed distribution, we implemented a stratified down-sampling strategy. For each unique escape score, we established a maximum retention threshold (e.g., 100 samples per value). Classes exceeding this threshold were randomly down-sampled, while those below were retained in their entirety.

Target Transformation: A log transformation was applied to the escape fractions ($y = \log(x)$) to stabilize the training objective. The processed dataset was partitioned into an 80% training set and a 20% validation set. The final independent validation set comprised 80 samples.

S2.3.2 Results

Data Distribution: Despite the stratified down-sampling strategy, the validation set still exhibits noticeable class imbalance, as shown in Table S2. Specifically, 73.8% of samples (59/80) remain as low-escape mutations with near-identical values. This persistence of imbalance reflects the underlying biological reality that the vast majority of single-point mutations do not substantially disrupt antibody binding.

Table S2: **Distribution of validation samples by escape level.** Characteristics of the 80 validation samples used for independent testing.

Escape Level	n	Percentage	Actual Score Range
Low (escape $<$ 0.08)	59	73.8%	-2.83 to -2.54
Medium-High (escape $\geq$ 0.08)	21	26.2%	-2.21 to -0.01

Quantitative Performance: While the overall Pearson correlation reached 0.53 (Table S3), this value should be interpreted in the context of the imbalanced distribution. For the medium-to-high escape subset (n=21)—where actual values show meaningful variation—the model achieved a Pearson r of 0.64 ($p = 0.002$). The model also demonstrated strong discriminative capability in identifying escape mutations (escape $>$ 0.37), achieving an **AUC-ROC of 0.85**.

Table S3: **Correlation metrics for the validation set.** Performance across all samples vs. the medium-high escape subset.

Metric	All Samples (n=80)	Medium-High Escape (n=21)
Pearson r	0.53 ($p < 0.001$)	0.64 ($p = 0.002$)
Spearman ρ	0.35 ($p < 0.01$)	0.54 ($p = 0.011$)

Limitations: Key limitations include: (1) persistent data imbalance influencing overall correlation metrics; (2) escape fractions reflect FACS-based selection rather than pure thermodynamic K_D; (3) the small validation set (n=80) limits statistical power. Nevertheless, the

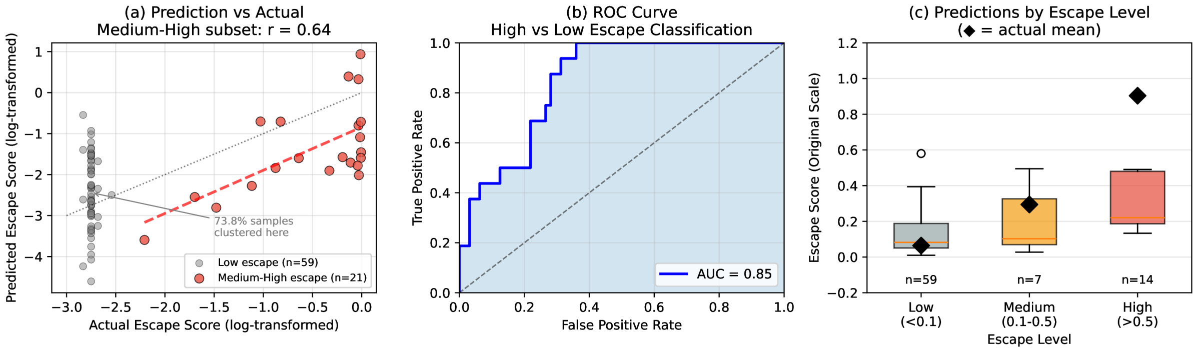

Figure S4: **Validation results on 7KMG (LY-CoV555).** (a) Scatter plot of predicted vs. actual log-escape scores; (b) residual distribution; (c) predictions stratified by escape level.

statistically significant correlation in the high-escape range supports the model's utility in prioritizing mutations for antibody engineering.

S2.4 KAN vs MLP Ablation Study

To justify the choice of Kolmogorov-Arnold Networks (KAN) over traditional multilayer perceptrons (MLP) for residue-level feature compression, we conducted an ablation study on both benchmark datasets.

Table S4: **KAN vs MLP ablation on sdAb-DB and AbBind.** Replacing KAN with a 2-layer MLP (2560→256→1, ReLU activation) consistently degrades performance across both datasets.

Dataset	Feature Compressor	MAE	RMSE	R^2	PCC
sdAb-DB	MLP (2-layer)	0.178	0.258	0.570	0.747
	**KAN (ours)**	**0.174**	**0.253**	**0.586**	**0.760**
AbBind	MLP (2-layer)	0.141	0.241	0.619	0.628
	**KAN (ours)**	**0.136**	**0.233**	**0.641**	**0.649**

The results demonstrate that KAN consistently outperforms MLP on both datasets. On sdAb-DB, replacing KAN with MLP increases MAE by 2.3% (0.174→0.178) and decreases PCC by 1.7% (0.760→0.747). On AbBind, MLP leads to a 3.7% increase in MAE (0.136→0.141) and a 3.2% decrease in PCC (0.649→0.628). These results empirically validate that KAN's learnable spline-based activations are better suited for the highly nonlinear mapping from pLM embeddings to interaction potentials.

References

[1] Emily E. Wilton, Michael P. Opyr, Senthilkumar Kailasam, Ronja F. Kothe, and Hans-Joachim Wieden. sdab-db: The single domain antibody database. *ACS Synthetic Biology*, 7(11):2480–2484, 2018.

[2] Sarah Sirin, James R. Apgar, Eric M. Bennett, and Amy E. Keating. Ab-bind: Antibody binding mutational database for computational affinity predictions. *Protein Science*, 25(2):393–409, 2016.

[3] NaturalAntibody. Indi - nanobody db. `https://research.naturalantibody.com/nanobodies`, 2022. Accessed: 2024-10-11.

[4] Yoichi Kurumida, Yutaka Saito, and Tomoshi Kameda. Predicting antibody affinity changes upon mutations by combining multiple predictors. *Scientific Reports*, 10(1):19533, 2020.

[5] Ruofan Jin, Qing Ye, Jike Wang, Zheng Cao, Dejun Jiang, Tianyue Wang, Yu Kang, Wanting Xu, Chang-Yu Hsieh, and Tingjun Hou. Attabseq: an attention-based deep learning prediction method for antigen–antibody binding affinity changes based on protein sequences. *Briefings in Bioinformatics*, 25(4):bbae304, 07 2024.

[6] Qing Wang, Yuzhou Feng, Yanfei Wang, Bo Li, Jianguo Wen, Xiaobo Zhou, and Qianqian Song. Antiformer: graph enhanced large language model for binding affinity prediction. *Briefings in Bioinformatics*, 25(5):bbae403, 08 2024.

[7] Minghui Li, Yao Shi, Shengqing Hu, Shengshan Hu, Peijin Guo, Wei Wan, Leo Yu Zhang, Shirui Pan, Jizhou Li, Lichao Sun, and Xiaoli Lan. Mvsf-ab: Accurate antibody-antigen binding affinity prediction via multi-view sequence feature learning. *Bioinformatics*, page btae579, 10 2024.

[8] Juntao Deng, Miao Gu, Pengyan Zhang, Mingyu Dong, Tao Liu, Yabin Zhang, and Min Liu. Nanobody–antigen interaction prediction with ensemble deep learning and prompt-based protein language models. *Nature Machine Intelligence*, 6(12):1594–1604, Dec 2024.

[9] Tyler N. Starr, Allison J. Greaney, Adam S. Dingens, and Jesse D. Bloom. Complete map of SARS-CoV-2 RBD mutations that escape the monoclonal antibody LY-CoV555 and its cocktail with LY-CoV016. *Cell Reports Medicine*, 2(4):100255, 2021.
